# Vaccine Virus Depot

**Published:** 1848-06

**Authors:** H. M. Congar

**Affiliations:** Committee


*Vaccine Virus Depot.*—The Buffalo Medical Association, at the meeting in June, resolved to establish a vaccine virus depot, and the shop of Mr. J. A. Matthews, apothecary, was by vote of the Society selected as the depot.

The regulations of the depot are contained in the following report of the committee on the subject , adopted by the association, and which we publish by request of the members present at the meeting.

Gentlemen:—Your committee, submit the following report, on the subject of Vaccine Virus, for your consideration: —

To us, the best method of preserving vaccine virus in this climate, appears to be, to surround the crust with tissue paper, and then envelope the whole in bees-wax.

We would reccommend the following plan, as a good method, if not the best, for securing, to the profession of our city, and perhaps to this region also, a *pure and efficient ariicle in abundance;* viz:

To establish with some druggist of our city a vaccine depot in which may be deposited the best virus, and *that only,* and which shall be under the following regulations:—1st, Physicians may with the druggist you may be pleased to select, deposite crusts to any amount in number, which shall be placed to their credit, at one dollar per crust. 2d. All persons taking virus from the depot, shall be charged by the druggist one dollar per crust. 3d, The money received by the druggist for virus sold, shall be his property as a remuneration for his trouble of conducting the establishment according to your dictation. 4th, Physicians having an account in the vaccine depot, shall not be allowed a transfer of credit from it to any other account they may at any time have with said druggist. 5th, Physicians, depositing virus in the depot, shall, with *every crust,* give to the druggist, the name, age, sex, and constitution of the individual from whom it was taken, the day it came off and *their own names.* 6th, The druggist shall keep a record of every particular thus given, numbering the detailed description of every crust, and placing a like number on the crust described. 7th, The druggist shall give in writing the description thus recorded, to every person purchasing virus, and shall allow no crust to leave the depot, without its accompanying description. 8th, Physicians shall under no *ordinary* circumstances, be allowed, under penalty of a vote of censure from this Society, to supply their brethren of the profession, or any other individuals, with vaccine virus for the purpose of *vaccination,* while there is a supply in the depot.

H. M. CONGAR, *Committee.*

